# Boon and bane of remission induction with rituximab in ANCA-associated vasculitis: lessons learned from the RAVE-ITN follow-up study

**DOI:** 10.12861/jrip.2014.05

**Published:** 2013-11-02

**Authors:** Andreas Kronbichler

**Affiliations:** ^1^Medical University Innsbruck, Department of Internal Medicine IV, Nephrology and Hypertension, Innsbruck, Austria

**Keywords:** ANCA-associated vasculitis, Rituximab, Remission

**Implication for health policy/practice/research/medical education:**

Rituximab has emerged as an alternative in the induction of remission in ANCA-associated vasculitis. Recent studies revealed a non-inferiority of a single dose rituximab in addition to a six month course of steroids compared to standard therapy after a follow-up period of 18 months. The results of the RAVE-ITN trial are encouraging and a special cohort of patients might benefit from this protocol.

Randomized controlled trials have revolutionised the treatment options in small vessel vasculitis. From large genome wide association studies we have learned that granulomatosis with polyangiitis (Wegener’s) and microscopic polyangiitis are distinct diseases (1). These findings have not been introduced in the design of newer clinical trials examining the efficacy of immunosuppressive therapies.

Rituximab (RTX), a monoclonal chimeric antibody targeting CD20 bearing B-cells, has been approved by the FDA and the EMEA for induction of remission in ANCA-associated vasculitis. The trials leading to the approval have been published with the aim to show non-inferiority of RTX when compared to cyclophosphamide (CYC). Both studies, the RAVE-ITN trial (2) and the RITUXVAS trial (3), included a divergent study population. In the RAVE-ITN trial patients had a well preserved renal function at the time of study enrolment (2), whereas patients in the RITUXVAS trial had severe renal insufficiency (3). Whereas RAVE-ITN recruited nearly 100 patients in each treatment arm (2), RITUXVAS assigned 33 patients to the treatment arm and 11 patients to the control arm (3). In RITUXVAS, patients received an initial course of CYC prior to receiving RTX infusions (3). Both trials reported distinct remission rates. In the RAVE-ITN trial, remission after six months of follow-up could be achieved in 64% of patients treated with RTX, while 53% of the patients receiving CYC were in remission. The primary efficacy end point was defined as a disease activity consistent with remission, which is defined as a BVAS [Birmingham vasculitis activity score (4)] of zero as well as a completion of prednisone tapering (2). RITUXVAS assessed prolonged remission rates at month 12 of follow up. There was no significant difference in the remission rate between both groups (76% in the RTX group and 82% in the CYC group) (3). In the RAVE-ITN trial subgroup analysis revealed that patients in the control group with newly diagnosed ANCA-associated vasculitis achieved higher remission rates than patients with RTX therapy (63% vs. 61%), although these differences did not reach significance (2). One has to emphasize the fact that a large proportion of patients with relapsing disease were included in the RAVE-ITN trial. Of these, a majority of patients in the control group received CYC to induce remission before. In the interpretation of these data, we have to be aware that these patients have failed to achieve a sustained remission following induction therapy. Thus, inclusion of these patients may have led to a selection bias.

The RAVE-ITN study population was further followed over a period of 18 months. The results revealed that a single course of RTX along with a 6 month regimen of steroids is as effective as CYC followed by azathioprine (AZA, standard of care) for the induction and maintenance of remission in ANCA-associated vasculitis. Remission could be maintained in 39% of the patients receiving RTX and in 33% of patients with CYC followed by AZA (5). These results are somehow surprising and need to be discussed in detail. The remission rates reported in the initial RAVE-ITN trial and in the follow-up period are surprisingly low and are by far exceeding expectations. In contrast, we learned from the CYCLOPS trial that approximately 20% of the recruited patients in the daily oral and 40% in the pulse intravenous CYC group relapse over a follow-up period of 4.3 years (6). Patients in the RAVE-ITN trial assigned to the control group received daily oral CYC therapy with a dosage of 2 mg per kilogram body weight with adjustment to the leukocyte count as well as to renal function (2). Within a period of 12 months, the remission rate dropped from 53% to 33% in the RAVE-ITN study (2,5). These findings warrant further observation to clarify the differences observed in both large trials. In addition, sparing of steroids is one of the most significant outcome parameter in nearly all autoimmune disorders. A recent meta-analysis from diverse trials revealed that early discontinuation of steroids is associated with a significant higher rate of relapses in ANCA-associated vasculitis (7). Thus, the attempt to completely discontinue steroids after a treatment period of 6 months seems to be at least partially responsible for the differences observed in the maintenance of remission between the above mentioned trials.

Reduction of relapses is one of the main outcomes relevant in daily clinical practice. Strategies with the aim to reduce relapses have been designed, but to date the rate of prolonged remission in ANCA-associated vasculitis is still unsatisfactory. More recently, a single center study revealed an efficacy of repeated RTX applications to maintain remission in ANCA-associated vasculitis. Interestingly, 73% of patients who received a single course of RTX to induce remission had a relapse over a period of 2 years, whereas 88% of the patients with RTX infusion on a regular basis (initial dose 2x1 g, followed by the infusion of 1 g every 6 months) had sustained remission. Unfortunately, patients receiving RTX maintenance in this study showed a trend to relapse after cessation (8). The efficacy of maintenance infusions was also supported by a recent work reporting on 28 patients with relapsing ANCA-associated vasculitis (9). Clinical trials with the aim to compare RTX with other common maintenance strategies are underway and will illustrate whether RTX should be used in this indication or not. One has to be aware that a prolonged RTX application may lead to a de novo or sustained hypogammaglobulinemia (10), potentially necessitate the use of rescue intravenous immunoglobulins or facilitating serious infectious complications.

Taken together, the RAVE-ITN trial was important to approve RTX as treatment strategy to achieve remission in ANCA-associated vasculitis. The single dose strategy together with no maintaining immunosuppression may be an option for a small collective, i.e. elderly patients with co-morbidities when prolonged immunosuppression may increase the overall risk for mortality. RTX is increasingly used in the therapy of autoimmune disorders (11) and future will tell us whether we can use B-cell depletion in the maintenance of remission in ANCA-associated vasculitis as well. The existing evidence reveals a striking reduction in the relapse rate when compared to standard immunosuppressive strategies used in this indication.

## Author’s contribution

AK is the single author of this manuscript.

## Conflict of Interests

Dr. Kronbichler received unrestricted research grants from GlaxoSmithKline and Roche/Genentech.

## Ethical considerations

Ethical issues (including plagiarism, informed consent, misconduct, double publication and redundancy) have been completely observed by the authors.

## Funding/Support

None declared.
